# Competences for implementation science: what trainees need to learn and where they learn it

**DOI:** 10.1007/s10459-020-09969-8

**Published:** 2020-05-05

**Authors:** Marie-Therese Schultes, Monisa Aijaz, Julia Klug, Dean L. Fixsen

**Affiliations:** 1 Department of Developmental and Educational Psychology, Faculty of Psychology, University of Vienna, Universitaetsstrasse 7, 1010 Vienna, Austria; 2 Department of Maternal and Child Health, Gillings School of Global Public Health, University of North Carolina at Chapel Hill, CB #7445 Rosenau, Chapel Hill, NC 27599-7445 USA; 3 Salzburg University of Education, Akademiestrasse 26, 5020 Salzburg, Austria; 4 AIRN Active Implementation Research Network, 6707 Glen Forest Drive, Chapel Hill, NC 27517-8647 USA

**Keywords:** Competence, Continuing education, Educational psychology, Evidence-based practice, Implementation practice, Implementation research, Implementation science, Knowledge transfer, Mixed methods, Training

## Abstract

Education in implementation science, which involves the training of health professionals in how to implement evidence-based findings into health practice systematically, has become a highly relevant topic in health sciences education. The present study advances education in implementation science by compiling a competence profile for implementation practice and research and by exploring implementation experts’ sources of expertise. The competence profile is theoretically based on educational psychology, which implies the definition of improvable and teachable competences. In an online-survey, an international, multidisciplinary sample of 82 implementation experts named competences that they considered most helpful for conducting implementation practice and implementation research. For these competences, they also indicated whether they had acquired them in their professional education, additional training, or by self-study and on-the-job experience. Data were analyzed using a mixed-methods approach that combined qualitative content analyses with descriptive statistics. The participants deemed collaboration knowledge and skills most helpful for implementation practice. For implementation research, they named research methodology knowledge and skills as the most important ones. The participants had acquired most of the competences that they found helpful for implementation practice in self-study or by on-the-job experience. However, participants had learned most of their competences for implementation research in their professional education. The present results inform education and training activities in implementation science and serve as a starting point for a fluid set of interdisciplinary implementation science competences that will be updated continuously. Implications for curriculum development and the design of educational activities are discussed.

## Background

### Introduction

Implementation science, “the scientific study of methods to promote the systematic uptake of research findings and other evidence-based practices into routine practice, and, hence, to improve the quality and effectiveness of health services and care” (Eccles and Mittman 2006, p. 1), has become a highly relevant topic in the health sciences. Clinical practice often considerably lags behind research findings, leading to poor health outcomes and an ineffective use of resources (Kien et al. 2018). Therefore, numerous frameworks describe how the transfer of scientific knowledge to health practice and the implementation of evidence-based interventions can be carried out systematically (e.g., Damschroder et al. 2009; Fixsen et al. 2005; Proctor et al. 2011). The complexity of implementation frameworks shows that carrying out and studying implementation projects requires a comprehensive set of knowledge and skills. These imply not only an understanding of implementation theory and research, but also skills that are necessary for building constructive partnerships with project stakeholders in the health system.

As a good example of systematic implementation practice and research that is based on a scientific framework, Blanchard et al. (2017) describe how they improved the implementation of comprehensive medication management in primary care practices across the United States. Implementation capacity building in this project included needs and readiness assessments for each site, an identification of program champions, practitioner coaching as well as the installation of improvement cycles and communication structures between practice and policy. Implementation research comprised developing fidelity measures and assessing fidelity concerning adherence to the intervention content, contextual support for the intervention and the practitioners’ competences at practice sites.

For professionally educating health specialists in also becoming implementation specialists, it is essential to define the necessary competences for research and practice in implementation and to create opportunities for training on respective knowledge and skills. Embracing the interdisciplinary nature of the field, perspectives from various disciplines that comprise implementation science (e.g., health sciences, social work, education) should be integrated in the definition of competence profiles. Furthermore, with implementation science being primarily a subject for continuing education of trainees from various backgrounds, trainees’ pre-education and experiences should be acknowledged in training concepts. Moreover, as a prerequisite for competence-based teaching, the concept of “competence” needs to be theoretically informed and consistently understood among experts who define competences for education in implementation science.

### Approaches for education and training in implementation science

In recent years, the field of professional education in implementation science has grown rapidly (Meissner et al. 2013; Proctor et al. 2013; Straus et al. 2015). Having been predominantly a topic for short-term training or isolated classes in university curricula, implementation science has become the center of several certificate programs at universities across the globe (Osanjo et al. 2016; Chambers et al. 2017; Ullrich et al. 2017). Hence, there is an increasing number of opportunities for students and professionals in healthcare to advance their knowledge and skills in implementation research and practice. Still, education and training in implementation science do not follow particular competence models that define what trainees in the field need to learn.

Implementation science is an evolving interdisciplinary field with a variety of different procedures, frameworks and terminologies (Durlak and DuPre 2008; Meyers et al. 2012; Tabak et al. 2012; Moullin et al. 2015). Hence, it is not obvious what the topical foci of respective educational curricula should be. Topics for implementation science classes have previously been prioritized based on implementation frameworks (Straus et al. 2011), surveys of expert groups (Moore et al. 2018; Tabak et al. 2017) or interviews with early-stage investigators (Stamatakis et al. 2013). Moreover, implementation experts, students and teachers have rated predefined competences according to their importance (Ullrich et al. 2017), skill levels (Padek et al. 2015) and their difficulty of being incorporated into a training curriculum (Tabak et al. 2017). The groups who have defined educational contents were mostly focused on a specific discipline and concentrated on a particular geographical area. Accordingly, competence profiles for implementation science that are created by diverse, international and multidisciplinary groups are still missing. The present study seeks to address this gap by presenting an international, multidisciplinary perspective on education in implementation science.

Another gap in research on education in implementation science is that it has not addressed trainees’ previous knowledge or diverse learning sources so far. With implementation science being an interdisciplinary field, the audience for professional education and training comes from various backgrounds with highly differing pre-education, experiences and training needs (Chambers et al. 2017; Proctor and Chambers 2017). Depending on their educational backgrounds, students of implementation science training might already have advanced research knowledge and skills but are seeking to improve their knowledge of implementation frameworks, or vice versa. Training needs might also differ for trainees seeking education in implementation practice or research. However, with few exemptions (e.g., Moore et al. 2018), most implementation science courses merely focus on implementation research so far (e.g., Ullrich et al. 2017) while there is little professional education in implementation practice (Proctor and Chambers 2017). The present study aims at contributing to an understanding of implementation experts’ sources of expertise and training needs while focusing on competences for both implementation practice and research.

### A theoretical basis for implementation science competences

In medical education—just as in many other educational contexts—there exist multiple definitions and conceptions of the term “competence” (Fernandez et al. 2012). In order to obtain clear definitions of implementation science competences, we integrate our study into an educational framework that also clearly defines what competences constitute. As competences have been a major research focus in educational psychology in recent years (Koeppen et al. 2008), our competence approach relates implementation science to educational psychology (Fixsen et al. 2016).

With the emphasis on products of educational processes, educational psychology defines competences as “context-specific cognitive dispositions that are acquired and needed to successfully cope with certain situations or tasks in specific domains” (Koeppen et al. 2008, p. 62; Leutner et al. 2017, p. 2) or “abilities to do something successfully or efficiently based on a range of knowledge and skills” (Bergsmann et al. 2018, p. 2). Accordingly, competence based medical education is “an approach to preparing physicians for practice that is fundamentally oriented to graduate outcome abilities and organized around competencies derived from an analysis of societal and patient needs. It de-emphasizes time-based training and promises greater accountability, flexibility, and learner-centredness” (Frank et al. 2010, p. 636), “(…) making it an individualized approach that is particularly applicable in workplace training” (ten Cate 2017, p. 1).

In a systematic review, Fernandez et al. (2012) found that most authors in medical education journals agree on knowledge and skills as constituting elements of competences. Other important characteristics of competences are that they refer to a specific domain and context and, most important for competence development, that they can be improved by training and deliberate practice (Koeppen et al. 2008; Shavelson 2013; Bergsmann et al. 2015). Often the terms “competence” and “competency” are used synonymously. However, the term competence is the broader, more holistic term (Blömeke et al. 2015), which seems to be more suitable for educational psychology as well as for implementation science.

Competence research has become especially important in educational psychology for implementing competence-based teaching in (higher) education. For that purpose, competence profiles for professionals working in particular fields as well as methods for assessing competences are needed. However, although competence-based curricula have a long tradition in health sciences education, aligning teaching and learning activities with competence profiles remains a challenge (Morcke et al. 2013). Accordingly, the implementation of competence-based teaching in higher education should be systematically guided and supported (Bergsmann et al. 2015). By relating to competence research from educational psychology, the present study aims at contributing a compendium of improvable and teachable competences for implementation practice and research. This compendium can build the basis for the development of education and training curricula in implementation science as well as for their evaluation and continuous improvement.

## The present study

The present study contributes to the advancement of education in implementation science as an evolving field within the health sciences by following two aims: The first aim is to compile a competence profile for conducting implementation practice and research that is based on educational psychology and provides a multidisciplinary view on implementation science competences. The second aim is to gain information on implementation practitioners’ and researchers’ existing competences and to explore where they have acquired their most helpful competences in order to assess common training needs and competence sources.

The present study comprises a survey of an international, multidisciplinary group of implementation practitioners and researchers. In order to get a better understanding of the participants’ positions and experiences, we followed a predominantly qualitative approach in data collection that explores the participants’ own verbalizations of competences. Using a concurrent triangulation mixed methods design (Creswell et al. 2003), we combined qualitative content analyses with descriptive statistics in data analysis.

## Methods

### Participants

We invited all registered participants of the Global Implementation Conference 2017 to participate in the study via an online survey. The conference had taken place in Toronto, Canada, in June 2017 and had hosted 514 international implementation researchers and practitioners from a variety of disciplines. The link to the survey was sent out by the conference organizers in August 2017, with two reminders 1 and 2 weeks after the initial invitation.

### Ethics approval and consent to participate

The study was reviewed by the Office of Human Research Ethics of the University of North Carolina at Chapel Hill and was determined to be exempt from further review. Participation in the study was voluntary and anonymous.

### Measures

The survey comprised demographical items on the participants’ area of work (e.g., practitioner, researcher) and their field of expertise (e.g., health, education). It was possible to select multiple answers and to provide other, open answers. Also, participants were asked to indicate their country of origin using a drop-down menu.

At the beginning of the survey, we provided the definition of competences by Bergsmann et al. (2018), emphasizing that competences are comprised of both knowledge and skills (see also Fernandez et al. 2012). An example from the implementation field followed the definition:

“We define competences as abilities to do something successfully or efficiently based on a range of knowledge and skills. An exemplary competence for both implementation research and practice would be ‘Defining the term implementation in a way which is understandable to the respective audience’.” The definition was shown on all following pages as a footnote.

Then, we asked participants to name competences that are helpful for conducting implementation practice (part A) and implementation research (part B) in several steps. The steps were identical in both parts of the survey. We framed implementation practice as “implementation capacity building”, as we assumed this wording to be more helpful when thinking of specific competences. In step one, we presented a vignette, asking participants to imagine they would hire a new co-worker who would be responsible for implementation capacity building (in part A) or implementation research (in part B). The participants were asked to name up to five competences that they would look for in applicants (*general competences*).

In step two, we asked the participants to indicate whether they had ever conducted implementation capacity building or implementation research themselves. If the answer was yes, they were asked to name up to five competences that had been helpful to them doing either of these activities (*personal competences*). In step three, the participants were presented with their personal competences and asked where they had acquired them (*competence sources*). They could assign each competence to “professional educational background”, “additional training” or “self-study or on-the-job experience”. Finally, the participants could name up to five competences that they would still like to expand or acquire.

The survey was piloted with a multidisciplinary group of eight implementation experts (from behavioral health, public health, pharmacy, psychology and education), whose feedback was integrated into the final version of the survey.

### Data analysis

Following a concurrent triangulation mixed methods design (Creswell et al. 2003), we integrated descriptive statistics in qualitative analyses of our data. For compiling the competence profile, we conducted a qualitative content analysis (Forman and Damschroder 2007) using MAXQDA Version 12 (VERBI Software 2017). Due to the exploratory nature of our research questions, we decided to follow an inductive approach of analysis that was based on our data material. In particular, one researcher, who is trained and highly experienced in qualitative data analysis, developed an inductive coding scheme based on the qualitative data and coded all open answers. A second researcher with considerable experience in qualitative research coded the entirety of open answers using the same coding scheme. Afterwards, the two researchers discussed differences in coding as well as the clarity of categories and refined the coding scheme together. Based on the final coding scheme, the first researcher coded all open answers again, which led to the final results.

In order to link the qualitative data on competences to their respective competence sources, which had been recorded as dichotomous variables, we transferred the coded data to SPSS Version 24 (IBM 2016). We analyzed the data descriptively by using frequencies and cross-tabs.

## Results

### Sample

A total of 82 delegates from the Global Implementation Conference 2017 participated in the survey, which corresponds to a return rate of 16 percent. Altogether, the 82 participants named 424 competences. The majority of the sample was from North America (66.7%), followed by Europe (19.8%), Australia (9.9%) and Africa (3.7%). The countries with the most participants were Canada (39.5%), the United States (27.2%) and Australia (9.9%). For defining their areas of work and fields of expertise, participants could provide multiple answers. Most participants defined themselves as researchers (46.3%) and/or practitioners (45.1%). Health (67.1%) and/or education (31.7%) were the most frequent choices for the field of expertise. Table 1 summarizes additional sample characteristics.

**Table 1:** Characteristics of study participants (n = 82)

Area of work^a^	Field of work
Researcher (46.3%)	
Practitioner (45.1%)	Health (67.1%)
Lecturer (8.5%)	Education (31.7%)
Student (8.5%)	Social work (20.7%)
Policy maker (7.3%)	Business (3.7%)
*Other*:	*Other*:
Consultant (6.1%)	Mental health (2.4%)
Implementation specialist (4.9%)	Child welfare (2.4%)
Trainer/TA provider/supervisor (4.9%)	Justice (2.4%)
Manager (2.4%)	Financial empowerment (2.4%)
Intermediary (2.4%)	

^a^Participants could provide multiple answers for area of work and field of work

Of the participants who defined themselves as researchers, most worked in health (30.5%), followed by education (17.1%). Similarly, most of the practitioners worked in health (29.3%) and/or education (20.7%).

### Competence profile for implementation practice and research

The qualitative content analysis of all named competences led to nine competence clusters relevant to implementation practice and implementation research: Implementation science knowledge and skills, setting knowledge and skills, program evaluation knowledge and skills, management knowledge and skills, research methodology knowledge and skills, academic knowledge and skills, collaboration knowledge and skills, communication knowledge and skills as well as educational knowledge and skills. Each competence cluster consists of three to eight sub-competences (Table 2), which all had a frequency of at least three responses.

**Table 2:** Competence profile for implementation practice and research resulting from the qualitative content analysis

Competence cluster	Sub-competences
Implementation science knowledge and skills	Knowledge of implementation theory and frameworks
	Ability to stay up to date with implementation research
	Specialized implementation knowledge and skills
	Systems knowledge
	Ability to apply implementation science knowledge
Setting knowledge and skills	Specific setting and content expertise
	Ability to identify setting characteristics
	Knowledge and understanding of policy
Program evaluation knowledge and skills	General program evaluation knowledge and skills
	Summative evaluation knowledge and skills
	Formative and process evaluation knowledge and skills
Management knowledge and skills	Project management knowledge and skills
	Self-management knowledge and skills
	Program development knowledge and skills
Research methodology knowledge and skills	General research knowledge and skills
	Ability to find and synthesize relevant information
	Knowledge of study designs
	General measurement and data analysis skills
	Qualitative research skills
	Quantitative research skills
	Mixed methods research skills
Academic knowledge and skills	Writing skills
	Publishing knowledge and skills
	Funding knowledge and grant writing skills
Collaboration knowledge and skills	General interpersonal skills
	Ability to network and build relationships
	Ability to collaborate and co-design
	Ability to work interdisciplinary and interculturally
	Knowledge and skills in stakeholder management
	Leadership skills
	Teamwork skills
	Ability to motivate and inspire
Communication knowledge and skills	General communication skills and strategies
	Ability to listen
	Ability to build shared understandings and agendas
	Visualization skills
Educational knowledge and skills	Ability to communicate complex ideas to varied audiences
	Presentation and facilitation knowledge and skills
	Adult education knowledge and skills
	Coaching knowledge and skills

When asked for the most helpful competences for implementation practice, participants named collaboration knowledge and skills most frequently. Apart from that, a high number of competences could be assigned to the implementation science knowledge and skills cluster.

For conducting implementation research, participants most frequently named research methodology knowledge and skills as the most helpful competences. The second most important competence cluster was implementation science knowledge and skills (see Table 3 for frequencies of all competence clusters).

**Table 3:** Proportional relevance of competence clusters for conducting implementation practice and implementation research

	Competences for implementation practice		Competences for implementation research	
	General competences (n = 180) (%)	Personal competences (n = 91) (%)	General competences (n = 106) (%)	Personal competences (n = 47) (%)
Implementation science knowledge and skills	20.6	19.8	16	14.9
Setting knowledge and skills	8.9	7.7	3.8	4.3
Program evaluation knowledge and skills	3.3	3.3	5.7	12.8
Management knowledge and skills	9.4	3.3	1.9	0
Research methodology knowledge and skills	13.3	12.1	32.1	27.7
Academic knowledge and skills	1.1	1.1	16	6.4
Collaboration knowledge and skills	22.8	27.5	10.4	17
Communication knowledge and skills	9.4	12.1	6.6	8.5
Educational knowledge and skills	11.1	13.2	7.5	8.5

General competences represent those that participants would look for in a new co-worker, whereas personal competences had been most helpful for their own implementation practice or research (n refers to the number of named competences)

Participants working in health named collaboration knowledge and skills (27.3%) and educational knowledge and skills (16.4%) as their most helpful personal competences for implementation practice. For participants working in education, competences in the implementation science (27.5%) and collaboration knowledge and skills cluster (25%) were the most important. Social work professionals most often named collaboration knowledge and skills (26.5%), followed by implementation science knowledge and skills (20.6%).

For implementation research, participants from the health sector named research methodology knowledge and skills (22.2%) and collaboration knowledge and skills (18.5%) as their most helpful personal competences. Participants from the education field most often named collaboration (23.3%) and implementation science knowledge and skills (16.7%). For participants with a social work background, research methodology (44.4%) and implementation science knowledge and skills (16.7%) were most important.

The most frequently named sub-competences that were perceived helpful for implementation practice were knowledge of implementation theory and frameworks (8.8%), the ability to network and build relationships (7.7%) and the ability to collaborate and co-design (6.6%). For conducting implementation research, the ability to find and synthesize relevant information, knowledge of study designs and general measurement and data analysis skills (each 6.4%) were indicated as the most helpful sub-competences.

Some responses that appeared several times were not attributed to a particular competence cluster, as they represent rather general characteristics than specific competences. The most frequent (for implementation practice and research combined) were flexibility (n = 14 mentions), the ability and willingness to learn (n = 10) and analytical thinking (n = 9).

### Competence sources and training needs

The participants had learned most of the competences that they found helpful for their implementation practice in self-study or by on-the-job experience, followed by their professional education and additional training. Concerning competences that were helpful for implementation research, participants had acquired most of them through professional education and self-study or on-the-job experience. Only a small percentage of implementation research competences have been acquired in additional training. Table 4 shows the frequencies of named competence sources for each competence cluster.

**Table 4:** Proportional relevance of professional education, additional training and self-study/on-the-job experience for the participants’ acquisition of competences for implementation practice and research (n refers to the number of named competences)

	Personal competences for implementation practice (n = 91)			Personal competences for implementation research (n = 47)		
	Professional education (%)	Additional training (%)	Self-study/on-the-job (%)	Professional education (%)	Additional training (%)	Self-study/on-the-job (%)
Implementation science knowledge and skills	38.9	27.8	33.3	42.9	42.9	14.3
Setting knowledge and skills	14.3	14.3	71.4	50	0	50
Program evaluation knowledge and skills	66.7	0	33.3	50	16.7	33.3
Management knowledge and skills	33.3	0	66.7	0	0	0
Research methodology knowledge and skills	63.6	27.3	9.1	76.9	7.7	15.4
Academic knowledge and skills	100	0	0	100	0	0
Collaboration knowledge and skills	16	4	80	0	0	100
Communication knowledge and skills	18.2	9.1	72.7	0	0	100
Educational knowledge and skills	16.7	41.7	41.7	50	0	50

Of those competences that participants would still like to expand or acquire for their implementation practice, most fall into implementation science knowledge and skills (33.3%), followed by collaboration (18.8%) and research methodology knowledge and skills (16.7%). More specifically, participants indicated that they would like to expand their specialized implementation knowledge and skills (17.9%) and their knowledge of implementation frameworks (5.4%). Apart from that, participants would like to acquire leadership skills (8.9%) as well as general research knowledge and skills and presentation and facilitation knowledge and skills (each 5.4%).

For their implementation research, most participants would like to expand or acquire competences attributed to research methodology (35.3%), program evaluation (17.6%) and communication knowledge and skills (17.6%). The most frequently named sub-competences were general program evaluation knowledge and skills (14.3%), quantitative research skills and the ability to build shared understandings and agendas (both 9.5%).

## Discussion

The present study aimed at advancing education in implementation science, and thus advancing the education of health professionals in how to implement evidence-based findings into health practice systematically. For that purpose, the present study compiled (1) a competence profile for implementation practice and implementation research and (2) competence sources and training needs for implementation science. In contrast to other competence studies in implementation science, we surveyed an international, multidisciplinary sample of implementation experts and theoretically based our study on educational psychology. Within this theoretical framework, competences are improvable and teachable and can be assessed in both trainees’ self-evaluations and evaluations of training programs and curricula.

The survey resulted in a competence profile including nine competence clusters. Although one highly relevant cluster comprised competences defining implementation science knowledge and skills, the remaining eight clusters mainly consisted of cross-sectional knowledge and skills. This result is similar to previous compendiums of implementation science competences (Tabak et al. 2012, 2017; Ullrich et al. 2017) that also emphasize the importance of a broad, interdisciplinary set of knowledge and skills. However, expanding the scope of other implementation science competence profiles, the present study explicitly assessed competences for both implementation practice and implementation research. When comparing results, competences from all nine clusters were relevant for both fields, but the priorities differed.

For implementation practice, competences belonging to the collaboration knowledge and skills cluster were named as most important. Certainly, these abilities are preconditions for successful implementation work in multi-professional and research-practice partnerships (Eriksson et al. 2014; Gagliardi et al. 2015). Well-functioning teams, communication and strong networks have been shown to facilitate the use of evidence-based practices in health (Jackson et al. 2018). Competences in areas such as communication, collaboration, and professional identity formation have also been named by the *ICBME* (international competency based medical education) *Collaborators* as desired outcomes for training in medical education (Frank et al. 2017). Furthermore, especially the importance of co-creation and constant feedback loops has often been emphasized by implementation experts (Fixsen et al. 2013; Metz and Albers 2014).

For conducting research on implementation, research methodology knowledge and skills, such as the ability to find and synthesize relevant information, were seen as the most important competences. These are also named in most other implementation science competence profiles (Padek et al. 2015; Ullrich et al. 2017; Tabak et al. 2017). Collaboration knowledge and skills were also listed frequently, which relates to an interview study with early career researchers (Stamatakis et al. 2013), who named “developing practice linkages” as one of the most important competences for conducting dissemination and implementation research. In this context, Luke et al. (2016) have shown that structured mentoring in implementation science significantly increases scientific collaboration.

The participants’ most important competence source for their implementation practice was their own on-the-job experience. This especially applied to their collaboration, communication and setting knowledge and skills. On the one hand, this result highlights the importance of practical experience for gaining implementation expertise. On the other hand, it indicates that collaboration and communication competences should become an integral part of implementation science curricula. This not only concerns teaching theoretical knowledge of how to build practice partnerships and models of communication but also applying collaborative teaching methods. The latter can include learning in peer groups, especially by jointly applying theoretical knowledge in real-life implementation projects as well as coaching and mentored supervision of practical implementation work. Methods like these include immediate feedback on practical activities while also providing a valuable professional network to trainees.

For building competences in implementation research, self-study and on-the-job experience were important as well, but participants had also acquired a considerable part of relevant competences in their professional education. The latter was especially an important source for the participants’ research methodology knowledge and skills. Hence, although knowledge of study designs and measurement issues as well as data analysis skills are important competences for conducting implementation research, most aspiring implementation experts might already be familiar with these topics from their former professional education. In order to get a valid estimation of these pre-experiences, a screening of participants’ competence levels in respective teaching contents of educational activities (Bergsmann et al. 2018) might be beneficial for both trainees and educators.

Since differences in learners’ pre-experiences are common in education and training in implementation science (Chambers et al. 2017), participants’ differing training needs can be met by building smaller groups within training sessions (Proctor and Chambers 2017). Another approach could be to offer implementation science curricula in a modular system, where trainees choose their modules according to the results of a competence assessment or the content’s relevance to their respective fields. Since a lot of continuing education activities in implementation science focus on particular topics, trainees can also arrange their educational curriculum according to their individual training needs. For that purpose, it is important to have an overview of learning and teaching resources in implementation science (Chambers et al. 2017), which can be easily accessed, e.g., in the form of a website (Proctor and Chambers 2017).

In terms of contents that institutions choose for their educational activities in implementation science, it makes sense to adapt general competence profiles to local needs, which also strengthens ownership among faculty (Hejri and Jalili 2013). This is especially relevant for the alignment of teaching and learning activities with desired learning outcomes. Bergsmann et al. (2015) provide a structured approach on how to define student competences for a curriculum in a participatory approach and how to align those with teaching methods and learning strategies. However, the benefit of competence profiles for teaching and learning in medical education still needs to be researched (Morcke et al. 2013). In this regard, an important aspect for discussion is the level of detail on which competences need to be defined, so that competence profiles are useful to both educators and trainees (Schultes et al. 2019).

### Limitations

The current study aimed at gaining an international perspective on competences for implementation research and practice. However, similar to previous research on the topic, the sample mostly consisted of participants from North America. Moreover, in comparison to the participant number of the Global Implementation Conference 2017, the return rate of surveys was rather low. Also, it was not possible to analyze whether respondents differed from non-respondents since the survey was anonymous, and the same sociodemographic data was not available for conference participants in general and those who participated in the survey. Further studies should anticipate this problem, for example, by including participant characteristics in the survey that are known for all potential participants. However, our sample named a total of 424 competences, which still provided us with a large database for our qualitative data analyses.

Future studies that validate and refine the competence profile from the present exploratory study might follow a quantitative approach of data collection and analysis that requires larger sample sizes. In this case, we suggest surveying participants from several different implementation science conferences or members from different implementation networks. The present study serves as a starting point for a fluid set of interdisciplinary competences. Education and training activities in implementation science should not only teach relevant foundations but also keep up with developments in the field (Chambers et al. 2017). Hence, competence profiles can only be seen as temporary, and the results from our present study should be regularly updated including participants with a broad distribution of regional and professional foci.

In order to support a specific notion of relevant competences, the survey items concerning implementation practice were framed as “implementation capacity building”. This wording might have triggered a specific set of implementation practice activities. For further research on competences in implementation science, we still consider it important to use a rather specific language, but the items should also be general enough to represent a large variety of implementation practice and research activities. Furthermore, it is important to refer to the knowledge and skill components of competences explicitly and to use examples referring to teachable and learnable competences. Overall, self-reported competences provide important information on participants’ experiences. Still, these should be complemented by other data sources, such as peer reports or surveys of other stakeholder groups from implementation studies (e.g., practice partners).

Since we followed an inductive approach of data analysis, the resulting sub-competences are closely aligned with the raw data and, as a consequence, not entirely consistent in their levels of abstraction. Although the competence clusters as a whole all comprise knowledge and skills in several sub-competences, not all of the sub-competences themselves consist of both knowledge and skill components. In order to create a more consistent competence profile for education and training in implementation science, the resulting sub-competences from this study can be aligned in their levels for further studies and used as a deductive coding scheme.

## Conclusions

Focusing on an emerging field in health sciences education, the present study compiled a competence profile for implementation science that includes a multidisciplinary perspective and is theoretically based on educational psychology. The competence profile comprises improvable and teachable competences that can be used for curriculum development in implementation science. Moreover, it can serve as a guide for choosing suitable training programs and for designing evaluations of education and training in implementation research and practice.

Since the present study showed that pre-experiences and competence sources differ among implementation experts, trainees’ pre-education should be considered when designing curricula and when allocating trainees to different training groups. With collaboration knowledge and skills being one of the most important competences for both implementation practice and research, the results of the present study also emphasize the importance of collaborative activities, such as learning in peer groups, coaching, or mentoring, in implementation science education. We encourage further research in competence development for implementation science that continuously updates the results of the present study and broadens our knowledge base for education in implementation science.
